# Effect of cup, syringe, and finger feeding on time of oral feeding of preterm neonate’s: a randomized controlled clinical trial

**DOI:** 10.1186/s41043-022-00336-4

**Published:** 2022-11-22

**Authors:** Parinaz Alinezhad Shebilouysofla, Manizheh Mostafa Gharebaghi, Niloufar Sattarzadeh Jahdi, Leila Abdoli Najmi, Sevil Hakimi

**Affiliations:** 1 Student’s Research Committee, Tabriz University of Medical Sciences, Tabriz, Iran; 2 Pediatric Health Research Center, Tabriz University of Medical Sciences, Tabriz, Iran; 3 Faculty of Nursing and Midwifery, Tabriz University of Medical Sciences, Tabriz, Iran; 4 Faculty of Nursing and Midwifery, Research Center of Psychiatry and Behavioral Sciences Tabriz University of Medical Science, Shariati Street, Tabriz, 5138947 Iran

**Keywords:** Cup feeding, Finger feeding, Full oral feeding, Methods of oral nutrition of preterm neonate, Syringe feeding

## Abstract

**Background:**

The oral nutrition is big challenge for preterm neonates. Since the best oral feeding method for preterm neonates is not yet known, the present study aimed to evaluate the effect of cup, syringe, and finger feeding methods on reaching the time of full oral feeding and weight gain among preterm neonates.

**Method:**

This randomized clinical trial study was conducted on 99 preterm neonate’s, born at 30–34 weeks gestation, admitted to the neonatal intensive care unit (NICU) of Al-Zahra and Taleghani Therapeutic-Educational Centers in Tabriz, Iran. Subjects were assigned into finger feeding (*n* = 33), cup feeding (*n* = 33), and syringe feeding (*n* = 33) groups in the allocation ratio of 1:1:1 using block randomization with a block size 6 and 9. They were studied in terms of reaching the time of full oral feeding and weight gain. The data were analyzed using SPSS/version21 software, and ANOVA, chi-square, and ANCOVA tests.

**Results:**

There was no significant difference in the mean score of reaching the time of full oral feeding among cup, finger, and syringe feeding groups (*p* = 0.652). The mean score of daily weight gain, oxygen saturation (SaO_2_), and heart rate after feeding was not significantly different among the three groups (*p* > 0.05). The effect of confounding variables, including birth weight and age, arterial oxygen saturation, and heart rate before feeding, was controlled.

**Conclusion:**

Based on the results, one of the cup, finger, and syringe feeding methods can be applied in the NICU, considering the staff’s proficiency in feeding neonates.

*Trial registration* IRCT20150424021917N11.

What is known

- Safe and efficient feeding method is one of the most important challenges in preterm newborns` life.
- Preterm neonate’s cannot coordinate breathing, Sucking, and swallowing, for oral feeding

What is new

- Results of this study showed that There was no significant difference in the reaching the time of full oral feeding among cup, finger, and syringe feeding methods.
- There was no difference in daily weight gain among neonates who fed with using of cup, Finger or syringe feeding methods

## Background

Acquiring safe and efficient feeding skills, is a challenging stage of life for preterm neonate. [1], which is one of the main and important components of emergency care for neonate’s [2, 3]. The factors influencing the effective feeding ability of preterm neonate’s include neurobehavioral maturity, physiological stability of the control of muscle tone, organization of behavioral state, swallowing, and coordinated breathing [4, 5]. Oral feeding requires the maturity of the sucking, swallowing, and breathing mechanisms. Preterm neonate’s cannot coordinate breathing, sucking, and swallowing, thanks to the dearth of physiological and neurological maturity [6]. Although the ability to suck and swallow is present by the 28 weeks gestation, the coordination of these abilities is not developed until the 32–34 weeks gestation. Therefore, neonate’s younger than 32 weeks of gestation cannot breastfeed or bottle feed efficiently and are fed by the gavage [7]. The appropriate nutrition for neonates is breastfeeding, which is achieved by successful sucking. However, preterm newborns fail to suck, due to the anatomical and physiological immaturity of the organs and systems and many problems they face. Therefore, alternative methods, such as using feeding tubes or supportive nutritional interventions, including cup, syringe, bottle, dropper, and finger feeding are suggested to prepare preterm neonate’s for breastfeeding [8, 9].

In cup feeding, the neonate is kept in a sitting or semi-sitting position with head and body coordination, as the rim of the cup is placed on his or her lower lip to lap or sip milk with forward movements of the tongue [10]. The World Health Organization (WHO) introduced cup feeding as a method of transfer or oral feeding complementation for preterm neonate’s, since it does not cause nipple confusion and does not affect the suction of preterm neonate’s [11–13]. The cup feeding allows the neonate to adjust the suction, control breathing, and swallow more easily, as it requires little energy [8].

The finger feeding is planed as another methodology within the nutrition transfer. During this technique, milk is transferred to the preterm neonate by suction through the nasogastric tube (NG tube) connected to a syringe attached to the little finger of the gloved hand, that is fastened in situ. Although this technique is widely used in different neonatal units, a few studies have been conducted on finger feeding, its indication and use, and its advantages and disadvantages [11, 12, 14, 15]. The sensory stimulation caused by the stiffness of finger is more like a nipple and facilitates the development of oral motor skills, which should be exist during breastfeeding [11, 15–17]. To our knowledge, there are few studies available on the use of syringe and only information exists on how to administer it. In finger feeding method, the milk is directed to the inner part of the neonate’s cheek and its piston is pressed only when the preterm neonate is sucking and not when swallowing or breathing [16].

The study results of comparing the effectiveness of the finger and syringe feeding methods indicated that the transition time to breastfeeding was significantly shorter and the weight gain was higher in the finger feeding group compared to the syringe feeding group [18].

In a study, neonates received gavage feeding at 26–32 weeks gestation were compared in terms of the time to start full oral feeding in the syringe and bottle feeding groups, and the time of transition to breastfeeding and the time of discharge were significantly short in the syringe group [19]. Owing to lack of sufficient evidence about comparing three mentioned nutrition method, this study was designed and carried out to response questions in this regard.

## Methods

### Study design

This randomized clinical trial study was done on 99 preterm neonate’s, born at 30–34 weeks gestation, admitted to the NICU of Al-Zahra and Taleghani Educational Centers in Tabriz. The sampling was carried out after obtaining the code of ethics from the Ethics Committee of Tabriz University of Medical Sciences (IR.TBZMED.REC.1399.819) and registering on the website of the Iranian Registry of Clinical Trials (IRCT, 20150424021917N11).

### Participants

Participants in this stusy were neonate’s at 30–34 weeks gestation with a stable clinical condition at the time of sampling, neonate’s swallow ability for two days, Apgar score higher than 7 at the 5th minute, and obtaining permission from the relevant neonatologist. The neonates with intraventricular bleeding and sepsis, neonate’s using continuous positive airway pressure (CPAP) or ventilator, and the presence of congenital malformation, Down syndrome, and neuromuscular diseases were not eligible. The researcher attended the selected centers for daily sampling, and subjects were selected using convenience sampling method.

### Recruitment, randomization and data collection

Before the start of the study, the researcher attended the educational-therapeutic centers and selected the neonate’s at 30–34 weeks. After checking the inclusion and exclusion criteria, their mothers were invited to participate in the briefing session. In the briefing session, more complete information was provided regarding the goals, study method, importance and benefits of participating in the study, then the initial registration of people interested in participating in the study was done and the check list of entry criteria for these neonate’s was completed. Informed written consent was provided to the mother of the neonate’s who met the conditions for inclusion in the study. The neonate profile questionnaire and the personal social profile questionnaire were completed. The researcher visited these centers daily and selected the eligible neonates as available. Participants were assigned into finger feeding (*n* = 33), cup feeding (*n* = 33), and syringe feeding (*n* = 33) groups in the allocation ratio of 1:1:1 by block randomization using the random allocation software (RAS) with a block size 6 and 9. For allocation concealment, the type of intervention was written on a piece of paper and placed inside sealed opaque envelopes that were numbered sequentially. The envelopes were opened by a non-involved person (a nurse working in the NICU) in sampling neonate’s who met the conditions to be included in the study, informed written consent was provided to the mother, the neonate and the mother profile questionnaires were completed.

### Data gathering tools

The data were collected using the neonate checklist, including heart rate, bradycardia, apnea, and oxygen saturation, neonate and mother’s socio-demographic characteristics questionnaire, including age, gender, neonate’s weight, birth grade, and mother’s age, Parental satisfaction with intervention questionnaire, which was measured using a Likert scale at the end of the intervention, and a checklist of adverse events, including any adverse events occurred to neonates during the intervention.

### Intervention

The neonate’s weight was measured at the beginning of the study and then, daily before and after feeding at a specific time in the morning shift using the scale available in the ward. Breast milk or pasteurized donor milk was used to feed the neonate. The amount of milk prescribed for each neonate was according to the protocol prescribed by the neonatologist. The feeding speed in the syringe feeding method was on average, 1.5 cc per minute [16]. Before starting the study, mothers were given the necessary training related to cup, finger, and syringe feeding methods. According to the hospital protocol, the gavage tube will not be removed from the neonate until reaching the full oral feeding. In this research, reaching the time of full oral feeding (oral feeding 8 times a day or two-thirds of the total number of feedings per day) is regarded as the primary outcome and weight gain, average heart rate, and arterial oxygen saturation as the secondary outcomes.

Oxygen saturation and heart rate were measured every day in the morning shift once at the beginning and then, at the end of breastfeeding. Further, the occurrence of apnea, bradycardia (heart rate reduction to less than 100 beats per minute), and chocking were monitored at each feeding time. The study continued until the neonate reached the independent oral feeding. Holding the syringe, finger or cup (disposable 50 ml plastic cup) on the neonate’s lips was considered as the onset and the completion of the milk prescribed by the neonatologist as the its termination. In all three groups, the neonate’s body was placed in a half-lying position with a 45-degree incline, and the head and neck were held with the other hand. At the end of the research, the intervention satisfaction questionnaire was provided to the mothers for completion.

### Outcomes

The primary outcome of this study was to compare the average time to oral feeding and weight gain in 3 groups for 5 days with the adjustment of birth weight using a newborn questionnaire.

The secondary outcome of the study was the comparison of the average heart rate, oxygen saturation, and the incidence of chocking, bradycardia, and apnea between the groups for 5 days of intervention. The mentioned variables were recorded once before and once immediately after the intervention in the morning shift for 5 days.

### Sample size

The sample size was calculated 33 subjects per group based on the study of Rahmani et al. [20]. Considering *m*_1_ = 5.1 (the mean duration to achieve full oral feeding in the syringe feeding group) and *m*_2_ = 4.0 (the mean day to reach full oral feeding in the cup feeding group), SD_1_ = 2.7, SD_2_ = 1.6, *α* = 0.05, *β* = 0.8, power = 80%, and two-sided hypothesis.

### Statistical analysis

The data were analyzed using SPSS/version_21_ software, ANOVA test was used to compare the quantitative variables of socio-demographic characteristics, and the chi-square was employed to compare the qualitative variables of socio-demographic characteristics among the three groups. In addition, ANCOVA test was applied to compare the time passed to reach oral feeding, weight gain, oxygen saturation, and heart rate after feeding. Birth weight, oxygen saturation, and heart rate before feeding were included into the statistical model as confounding variables. The chi-square test was used to compare side effects (chocking, bradycardia, and apnea) and mothers’ satisfaction with feeding methods by intervention groups. In all stages, the P-value was considered 0.50 and the data were analyzed using the Intention to treat method.

## Results

**Fig. 1:**
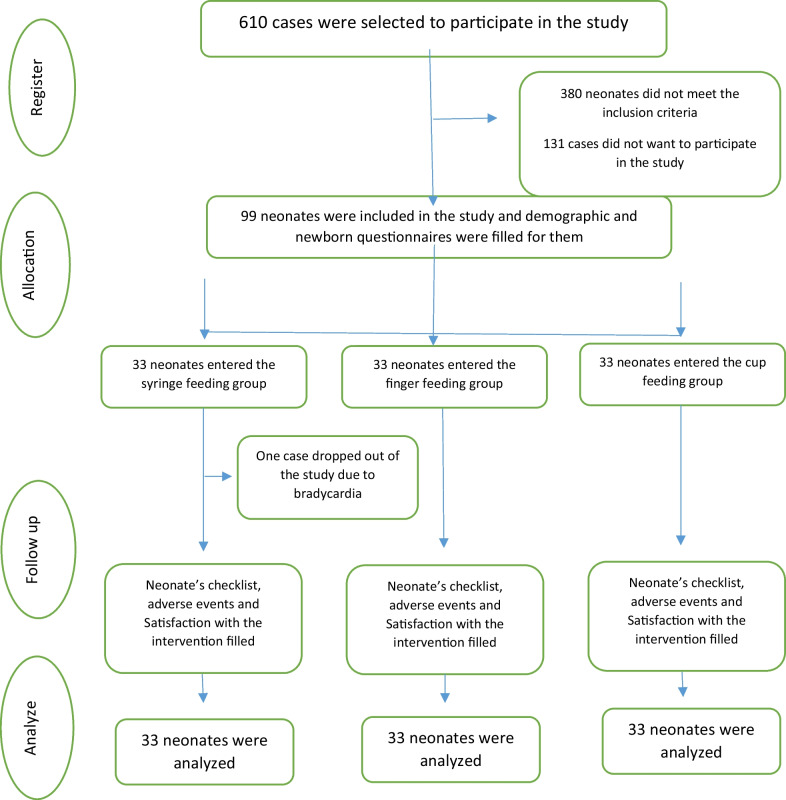
Study flowchart

This study was conducted from February to October 2021. The characteristics of 610 neonate’s were checked initially and 511 neonates were excluded from the study, due to not fulfilling the eligibility criteria (Fig. 1). Finally, a total of 99 preterm neonate’s were entered in the cup feeding (*n* = 33), finger feeding (*n* = 33), and syringe feeding (*n* = 33) groups. One neonate in the syringe feeding group was excluded from the study due to bradycardia. The mean (SD) age of the neonate’s was 30.6 (2.1) weeks and the birth weight was 1560.45 (432.23) gr.

Table 1 indicates the socio-demographic characteristics of neonate’s and mothers. The results of the ANOVA test demonstrated a significant difference in the neonates’ birth weight and age among the three groups. Tukey’s tests revealed that cup feeding neonates had significantly higher birth weight (*p* = 0.045) and age (*p* = 0.002) compared to finger feeding neonate’s.

**Table 1:** Individual and social characteristics of the study participants

Groups	Cup feeding	Finger feeding	Syringe feeding	*p* value **
Variable	*N* = 33Mean (SD)*	*N* = 33Mean (SD)*	*N* = 33Mean (SD)*	
Mother's age (years)	29.06(7.41)	27.24(4.82)	29.51(5.38)	0.268
Neonate’s age at recruitment (days)	13.36(11.19)	21.09(15.31)	18.51(13.82)	0.068
Gestational age (weeks)	31.48(1.87)	29.66(2.13)	30.84(2.19)	0.002
Neonate’s birth weight (grams)	1661.36(399.57)	1407.57(382.22)	1612.42(485.10)	0.040
*Mother’s job*				
Housewife	31(93.9)	31(93.9)	30(90.9)	0.858
Employed	2(6.1)	2(6.1)	3(9.1)	
*Education*				
Illiterate/elementary	3(9.1)	1(3.0)	3(9.1)	0.545
Middle school/high school	9(27.3)	5(15.2)	7(21.2)	
Diploma/University	21(63.6)	27(81.8)	23(69.7)	
*Gravid*				
1–2	22(66.7)	23(69.7)	21(63.6)	0.706
3–4	23(69.7)	10(30.3)	10(30.3)	
> 4	2(6.1)	0(0.0)	2(6.1)	
*Delivery*				
1–2	25(75.8)	28(84.8)	25(75.8)	0.580
3–4	8(24.2)	5(15.2)	8(24.2)	
*Delivery type*				
Vaginal	9(27.3)	6(18.2)	6(18.2)	0.580
Cesarean section	24(72.7)	27(81.8)	27(81.8)	
*Gender of the neonate*				
Female	19(57.6)	15(45.5)	15(45.5)	0.524
Male	14(42.4)	18(54.5)	18(54.5)	
*Receive breastfeeding education*				
Yes	33(100)	33(100)	33(100)	
No	–	–	–	–

*Mean (standard deviation) ** ANOVA test and chi-square test and chi-square test

Table 2 represents the time to reach oral feeding and weight gain during the study. The results of ANCOVA indicated no difference in the neonate’s weight among three groups after adjusting the effect of weight.

**Table 2:** Comparison of the time to reach full oral feeding and daily weight gain according to the study groups

Group	Time (day) Mean(SD)*	Weight (gram) Mean(SD)*	ADM(95%CI)¥	*p* value**
*Time to reach oral feeding*				
Cup feeding (*n* = 33)	6.27(5.03)			0.652
Finger feeding (*n* = 33)	9.12(6.53)			
Syringe feeding (*n* = 33)	7.96(5.17)			
*Comparison of groups*				
Cup with finger feeding			− 1.1(− 4.4 to 2.26)	0.212
Cup with syringe feeding			− 1.03(− 2.10 to 4.17)	0.652
Syringe feeding with finger			0.076(− 3.38 to 3.23)	0.325
*Daily weight gain (grams)*				
Cup feeding		14.31 (6.11)		0.127
Finger feeding		12.90 (4.7)		
Syringe feeding		15.55 (4.83)		
*Comparison of groups*				
Cup with finger feeding			1.13(− 2.10 to 4.37)	0.781
Cup with syringe feeding			− 1.19(− 4.1 to 1.72)	0.691
Syringe feeding with finger			2.32(− 0.93 to 5.58)	0.237

*Mean (standard deviation) ¥ Adjusted mean difference (95% confidence interval)

**ANCOVA test with adjustment of weight, gestational age and birth weight

Table 3 illustrates the average heart rate and oxygen saturation after feeding by the study groups. Based on the results of ANCOVA, there was no difference in neonates’ age at birth among three groups after adjusting the effect of weight. However, the heart rate of neonates in syringe feeding group was significantly higher than that in cup feeding group (*p* = 0.026).

**Table 3:** Comparison of oxygen saturation and heart rate after feeding by intervention groups

Group	Arterial oxygen mean(SD)*	Heart rate mean(SD)*	ADM(95%CI)¥	*p* value**
*Arterial oxygen*				
Cup feeding (*n* = 33)	96.60(1.38)			0.337
Finger feeding (*n* = 33)	96.12(0.90)			
Syringe feeding (*n* = 33)	96.16(1.92)			
*Comparison of groups*				
Cup with finger feeding			0.32(− 0.50 to 0.57)	0.998
Cup with syringe feeding			0.31(− 0.22 to 0.84)	0.406
Syringe feeding with finger			− 0.27(− 0.81 to 0.25)	0.508
*Heart rate*				
Cup feeding		142.11(9.47)		0.233
Finger feeding		145.28(8.48)		
Syringe feeding		145.05(6.37)		
*Comparison of groups*				
Cup with finger feeding			− 1.45 (− 3.39 to 0.48)	0.200
Cup with syringe feeding			− 3.60(− 5.55 to − 1.65)	0.200
Syringe feeding with finger			2.14(0.19 to 4.10)	0.026

*Mean (standard deviation) ¥ Adjusted mean difference (95% confidence interval)

**ANCOVA test with adjustment of oxygen saturation and heart rate from feeding

In the present study, no case of apnea was reported in any of the groups. In the syringe feeding group, one person suffered from bradycardia and 13, 11, and 14 cases of chocking occurred in the cup feeding, finger feeding, and syringe feeding groups, respectively.

## Discussion

The current study aimed to compare the cup, syringe, and finger feeding methods on reaching the time of full oral feeding and weight gain among the preterm neonate’s. To the best of our knowledge, no study has compared the effect of the cup, syringe, and finger feeding methods on reaching the time of full oral feeding and weight gain among preterm neonate’s. In most of the studies, two feeding methods have been compared and in some of them, three methods have been compared with other variables.

The mean duration to reach full oral feeding did not show a significant difference between the study groups, only the time to reach full oral feeding in the cup feeding group was slightly less than that in the other groups. Although the weight gain was slightly higher in the syringe feeding group, there was no significant difference among the three groups.

Nunes et al. [21] in a clinical trial study evaluated the provision of a diet with cup feeding and finger probe simultaneously with breastfeeding and indicated additional weight gain and longer length of hospital stay in the cup feeding group. The more weight gain was probably due to the longer length of stay in the hospital. There was no statistically significant distinction among the study groups in terms of oxygen saturation and heart rate.

In line with the results of the present study, the findings of the study of Mirjalili et al. [22] discovered that the mean weight of neonate’s and therefore trend of changes within the mean weight were not considerably completely different in the cup, finger, and dropper feeding methods (*p* = 0.25).

Achieving full oral feeding is an important step for preterm infants, given that it is an important criteria in order to discharge of newborn and shows the maturity and health of the preterm infant [23], Any delay in achieving this crucial physiological function will lead to delay in discharge from the neonatal intensive care unit and might result in growth failure, and poorer neurodevelopmental outcomes [24–26].

Çamur et al. [27] reported that the bottle and cup feeding methods were equally effective in reaching the time of full oral feeding and there was no statistically significant difference between the mentioned groups. However, the study results of Say et al. [19] illustrated that the transition time to full oral feeding was significantly shorter in syringe-fed preterm neonate’s compared to bottle-fed preterm neonate’s.

There is insufficient evidences comparing dietary transition techniques in respect to O2 Saturation and heart rate [21]. It is possible that the type of feeding of premature neonate’s affects heart rate fluctuations and arterial oxygen saturation. López et al. [28] in their study showed that O_2_ Sat was less than 85% after cup feeding. The authors emphasize that the probable fall of O_2_ Sat may be related to the vigorous attempt to suck the milk from the cup.

Araújo et al. [16] observed that oxygen saturation and heart rate variations observed before, during, and after feeding were within normal limits for both syringe and finger feeding methods. In addition, oxygen saturation increased between the moments before and after the syringe feeding.

Among the neonate’s feeding options, the cup feeding may be an easy method with a protracted history and a semipermanent feeding solution for those that cannot breastfeed [29], which may be used to supplement breastfeeding and minimize gavage exposure. The theoretical advantages of cup feeding include avoiding any confusion between the breast and the bottle, increasing the neonate’s sucking ability, and facilitating the neonate’s ability in self‐regulation and feed demand [8, 30]. Further, there are many benefits to cup feeding, including strengthened bonding, mother’s higher sense of control and confidence, the possibility to engage other family members in caring for the neonate, and freeing up the nursing staff when the mothers conduct health care [29]. The main and most important use of the cup feeding is to provide a safe artificial feeding method for preterm and low birth weight neonate’s until they become strong and grow up enough to exclusively breastfeed [31].

The finger probe method is emerged as an option to transfer nutrition, which is widely used in the various service routines as a suction training method or as a supplementary method. Finger probe method is used during feeding as an option when there is no good compatibility with the cup [8, 12, 13, 32]. Finger feeding is a safe method for preterm neonate’s, which can be recommended to accelerate the transition to breastfeeding, increase the rate of weight gain, and shorten the hospitalization period [18]. The evidence revealed that the finger feeding method requires more time and costs compared to the cup feeding method. Nevertheless, finger feeding method provides oral stimuli to the neonate’s, which is beneficial for suction training, maintaining alertness, and coordination of suction, swallowing, and breathing. The weight gain of finger-fed neonate’s is more than that of syringe-fed neonate’s. Given the advantages of finger feeding method in terms of achieving oral feeding of preterm neonate’s, more convenience, and shorter hospitalization time, especially in the gestational age below 34 weeks, finger feeding is considered as a desirable method [13, 17, 18, 33].

In the syringe feeding method, the milk was directed to the inner part of the neonate’s cheek and also the piston was ironed once only if when the preterm neonate was sucking and not when swallowing or breathing, and the rate was twenty cc per minute [16]. Although syringes are commonly used in neonatal wards, this method provides an anti-physiological stimulus regardless of the neonate’s desire to suck or reach [34].

In the present study, the level of mothers’ satisfaction with the intervention was slightly higher in the syringe feeding group. However, there was no statistically significant difference among the three groups. The same level of satisfaction indicates that all three feeding methods can be used for mothers in breastfeeding the preterm neonate’s. In the present study, 13, 11, and 14 cases of chocking occurred in the cup feeding, finger feeding, and syringe feeding groups, respectively, as there was no statistically significant difference among the three groups. However, chocking was slightly more in the syringe group and slightly less in the finger group compared to the other two groups. Given that, the milk flows continuously in the syringe feeding method and the neonate does not control the volume of milk entered in the oral cavity, more chocking probably occurred in the syringe feeding group for this reason.

### Strength

The randomized controlled trail study design is regarded as one of the strengths of this research.

### Limitation

The birth weight recorded in the newborn’s cases was entered in the socio-demographic characteristic questionnaire. Given that the birth weight was not measured by the researcher, this is considered as one of the limitations of this research. The small sample size in each group is another limitation of the present study. Further, 80% power was considered in the calculation of the sample size.

## Conclusion

Based on the results of the present study, the cup, syringe, and finger feeding methods had no remarkable difference on reaching the time of full oral feeding and weight gain of preterm neonate’s, as well as heart rate and arterial oxygen saturation after feeding. The level of mothers’ satisfaction with the cup, syringe, and finger feeding methods was not different in the three groups, and suffocation on milk in the syringe feeding group was slightly higher than that in the other two groups, which is negligible. Therefore, one of the cup, syringe, and finger feeding methods can be considered in NICU based on the staff’s proficiency in feeding neonates. However, conclusions should be made with caution, due to the small sample size.

## Data Availability

The datasets used and/or analyzed during the current study are available from the corresponding author on reasonable request.

## Abbreviations

NICU: Neonatal intensive care unit
ANOVA test: Analysis of variance test
ANCOVA: Analysis of covariance test
SaO_2_: Arterial oxygen saturation
IRCT: Iranian registry of clinical trials
WHO: World health organization
NG: Nasogastric
CPAP: Continuous positive airway pressure
ITT: The Intention to treat
